# Catheter Ablation of Life-Threatening Ventricular Arrhythmias in Athletes

**DOI:** 10.3390/medicina57030205

**Published:** 2021-02-26

**Authors:** Nicola Tarantino, Domenico G. Della Rocca, Nicole S. De Leon De La Cruz, Eric D. Manheimer, Michele Magnocavallo, Carlo Lavalle, Carola Gianni, Sanghamitra Mohanty, Chintan Trivedi, Amin Al-Ahmad, Rodney P. Horton, Mohamed Bassiouny, J. David Burkhardt, G. Joseph Gallinghouse, Giovanni B. Forleo, Luigi Di Biase, Andrea Natale

**Affiliations:** 1 Arrhythmia Service, Department of Medicine, Division of Cardiology, Montefiore Medical Center, Bronx, NY 10467, USA; nicolatarantinomd@gmail.com (N.T.); emanheimer@gmail.com (E.D.M.); dibbia@gmail.com (L.D.B.); 2 St. David’s Medical Center, Texas Cardiac Arrhythmia Institute, 3000 N. IH-35, Suite 720, Austin, TX 78705, USA; mitra1989@gmail.com (S.M.); Chintan.Trivedi@stdavids.com (C.T.); aalahmadmd@gmail.com (A.A.-A.); rodney.horton@gmail.com (R.P.H.); bassiounymd@gmail.com (M.B.); John.Burkhardt@stdavids.com (J.D.B.); jgallinghouse@gmail.com (G.J.G.); dr.natale@gmail.com (A.N.); 3 Brookdale University Hospital Medical Center, Brooklyn, NY 11212 USA; dra.deleon33@gmail.com; 4 Department of Cardiovascular/Respiratory Diseases, Nephrology, Anesthesiology, and Geriatric Sciences, Policlinico Umberto I, Sapienza University of Rome, 00185 Rome, Italy; michelefg91@gmail.com (M.M.); carlolavalle@yahoo.it (C.L.); 5 Department of Cardiology, Azienda Ospedaliera-Universitaria “Luigi Sacco”, 20057 Milano, Italy; forleo@me.com; 6 Department of Clinical and Experimental Medicine, University of Foggia, 71122 Foggia, Italy; 7 Interventional Electrophysiology, Scripps Clinic, La Jolla, CA 92037, USA; 8 Department of Cardiology, MetroHealth Medical Center, Case Western Reserve University School of Medicine, Cleveland, OH 44106, USA

**Keywords:** athletes, catheter ablation, sports cardiology, ventricular arrhythmia, ventricular tachycardia

## Abstract

A recent surveillance analysis indicates that cardiac arrest/death occurs in ≈1:50,000 professional or semi-professional athletes, and the most common cause is attributable to life-threatening ventricular arrhythmias (VAs). It is critically important to diagnose any inherited/acquired cardiac disease, including coronary artery disease, since it frequently represents the arrhythmogenic substrate in a substantial part of the athletes presenting with major VAs. New insights indicate that athletes develop a specific electro-anatomical remodeling, with peculiar anatomic distribution and VAs patterns. However, because of the scarcity of clinical data concerning the natural history of VAs in sports performers, there are no dedicated recommendations for VA ablation. The treatment remains at the mercy of several individual factors, including the type of VA, the athlete’s age, and the operator’s expertise. With the present review, we aimed to illustrate the prevalence, electrocardiographic (ECG) features, and imaging correlations of the most common VAs in athletes, focusing on etiology, outcomes, and sports eligibility after catheter ablation.

## 1. Introduction

Up-to-date prospective surveillances estimate that the incidence of sudden cardiac death/arrest (SCD/SCA) is ≈1:50,000 amongst professional athletes per year [1]. Considering that valvular and congenital cardiac defects, aortic dissection, myocarditis, and negative autopsy represent 40% of the findings, the net majority of life-threatening events in athletes is ascribable to primarily arrhythmogenic ventricular cardiac conditions, such as inherited/acquired cardiomyopathies (i.e., arrhythmogenic dysplasia (ARVD), idiopathic hypertrophy), coronary artery disease (CAD), and channelopathies [2]. The cardiac remodeling triggered by intense regular physical activity is equally responsible for the impairment of the electrical milieu [3,4]. Such remodeling sometimes configures peculiar electro-anatomical pictures for both the atrium and the ventricle [4,5,6,7,8]. Ventricular manifestations of rhythm disorders are common in professional and semi-professional athletes [9], some of which are either genuinely symptomatic or concerned for a possible non-eligibility to official competitions.

The choice of pharmacological treatment can be challenging because of possible low tolerance and pro-arrhythmic effects. In addition, similarly to physical detraining, antiarrhythmics can impact the athlete’s physical performance [10]. Therefore, more durable and short-term treatments like catheter ablation are necessary to overcome all these inconveniences, improve the symptoms, shorten the detraining time, and eventually restore professional sports eligibility. This review focuses on the role and benefit of catheter ablation of ventricular tachyarrhythmias in the athletes, describing the variety of underpinning common substrates for this subset of patients, highlighting the indications, the outcomes, and current gaps in the fields. 

## 2. Methods 

The present review was conducted using PubMed and Google Scholar. Search terms used were “sport” OR “sport cardiology” OR “athletes” OR “exercise” AND “ventricular arrhythmia” OR “ventricular tachycardia” OR “sudden cardiac death” OR “hypertrophic cardiomyopathy” OR “arrhythmogenic right ventricular dysplasia” OR “Brugada” AND “ablation” OR “catheter ablation” AND “heart” AND “electrophysiology” (Table 1). Web sites, including acc.org and escardio.org, were also assessed for relevant materials. References of the articles identified in this manner were also searched through to locate additional references that—not identified by the search strategy—might be useful for the purpose.

The search was limited to the relevant English-language publications in a period of time from 1990 to 2021.

## 3. Epidemiology and Features of VAs in Athletes

A significant limitation to the characterization of VAs in athletes lies in the high heterogeneity of the endpoints (premature ventricular complex (PVC), sustained vs. non-sustained ventricular tachycardia (VT)) and methods of screening (i.e., 12-lead ECG, Holter, exercise test, invasive study) used by different study groups over the time [2,11,12,13,14,15,16,17,18,19].

In a registry of 1644 competitive athletes, VAs were present in 2% [20]. A non-controlled sub-analysis of the same registry including athletes (98% males, age 30 ± 13 years) with an echocardiogram and cardiac magnetic resonance (CMR) negative for structural heart disease (SHD) showed that 10/13 (77%) presented sustained or non-sustained VT (3 and 7 patients, respectively). In comparison, only 3 (23%) had frequent PVCs (defined as >1000/24 h). Stress test ECG diagnosed the majority of VAs, and a left-bundle branch block morphology (LBBB) with an inferior axis (indicating a right ventricular outflow tract (RVOT) origin) was predominant in nearly 80% of the cohort, as opposed to a minority of patients with possible LV origin (right bundle branch block (RBBB) morphology of the VAs in the remaining three patients). 

Another prospective non-controlled analysis on 46 high-intensity athletes reported similar results, with a history of SCA in 2% of the cohort and a prevalence of LBBB morphology characterizing 80% of the VAs [21]. Of interest, the exercise stress test and electrophysiological study outperformed the Holter to detect sustained VT (≈35% vs. 2% of the total VAs recorded). 

From a case-control single-center study, it emerged that the prevalence of either frequent (>10 PVCs/hour) or complex (>1 couplet) VAs detected with the help of a 12-lead Holter did not differ between 288 athletes (*n* = 28, 10%) and 144 sedentary subjects (*n* = 13, 11%, *p* = 0.62). Moreover, it was unrelated to the type of sport, hours of training, and years of activity [22]. Similarly, non-sustained VT (>4 beats) occurred in 6 athletes vs. 3 (*p* = 1); on the contrary, isolated sporadic (1–10) PVCs were more commonly counted in the athletes (141 (49%) vs. 41 (28%), *p* = 0.001). Still in the same study, half of the VAs displayed LBBB morphology with an inferior axis in 38%, LBBB with a superior axis in 22%, followed by fascicular morphology (RBBB-like and QRS <130 ms) in 15%.

More recently, 12-lead ECG features of the clinical VT in a cohort of 57 endurance athletes undergoing ablation typically showed LBBB morphology in all of the patients [7]. However, the superior axis was expressed only in the group with sub-tricuspid scar (12/46 (26%) vs. 0/11), in comparison to the inferior axis that was seen in patients with epicardial right ventricle outflow tract scar (8/46 (17%) vs. 11/11 (100%) *p* < 0.001). Of note, six patients (13%) of the first group, but none of the other group, presented VF.

## 4. Arrhythmogenic Ventricular Substrate in Athletes

Clinical evidence regarding the epidemiology and the physiopathology of VAs in athletes has recently cleared several nebulous concepts derived from highly heterogeneous populations studied in the past. In fact, just in the last decade, it has been acknowledged that, as for the general population, the pattern of VAs and myocardial disease in athletes differs depending on the age, type, and years of training, as well as gender [23,24,25,26,27]. Environmental and ethnical factors can also be invoked, as European studies find a relative higher proportion of ARVD, compared to analysis carried in North America, where greater prevalence of hypertrophic cardiomyopathy has been reported [1,7,20,21].

An insight into the prevalence of different cardiac disorders amongst the athletes derives from a very recent analysis of young American competitive athletes who experienced SCD/SCA [1]. This study showed that hypertrophic cardiomyopathy and idiopathic left ventricular hypertrophy accounted respectively for 21% (*n* = 43) and 13% (*n* = 28) of the 331 episodes of cardiac arrest (more than a half eventually fatal). Coronary anomalies represented the second most common anatomical aberrancy (25%, *n* = 12); however, the etiology and distribution were age-dependent: Cardiomyopathies (hypertrophic, dilated, restrictive, non-compacted, arrhythmogenic dysplasia) being prevalent in the third decade of age (47% of the 34 college/professional athletes), and the anomalies of the coronary arteries predominating in the middle/high school age (27/157 vs. 1/34 of older athletes), confirming what has been previously documented [21,22].

Although the benefit of physical exercise should not be questioned, the effects on cardiovascular wellness are not linearly correlated with the intensity and duration of the training, as indicated by the observation that CAD is surprisingly prevalent amongst athletes, especially after the fifth decade [28,29]. A recent study on the prevalence and composition of subclinical CAD found a statistical prevalence of atherosclerotic plaques of any luminal irregularity (44.3% versus 22.2%; *p* = 0.009) in 152 endurance master athletes (70% male, age 54 ± 9 years) compared to 94 sedentary controls with a similar risk profile [30]. Late gadolinium enhancement (LGE) was seen in 15 (14.2%) male athletes compared with none of the controls (*p* = 0.004), featuring equally a non-ischemic epi-mid-myocardial (*n* = 8) and ischemic sub-endocardial (*n* = 7) scar pattern. Interestingly, although the authors did not focus on the arrhythmic profile in their cohorts, non-sustained VT was observed only in 9 male athletes—3 of which presented an ischemic pattern of LGE—but in none of the controls (*p* = 0.02). In addition, there was no difference between athletes and controls of the female gender.

Other investigations found that athletes present a specific myocardial disease attributable neither to ischemia nor to primary cardiomyopathies, but probably secondary to the heart physiology’s perturbations due to constant strenuous exercise. On a cohort of 288 competitive athletes with a variable number of VAs detected by Holter monitoring, only 3 of the 17 cases (20%) showing serious VAs (>500 isolated PVCs or runs, or exercise-induced VT) had documented non-ischemic (epicardial/mid-myocardial) focal LGE of the left ventricle (LV) [22]. Of note, the right ventricle (RV) appeared healthy in all of these patients who exhibited RBBB morphology of the VAs, in line with the imaging finding of exclusive involvement of the LV. Contrarily, according to other pivotal studies, the athletes’ arrhythmogenic substrate resides in the RV rather than the LV [7,20,21]. 

In an original single-center case series by Dello Russo et al., 10 (77%) of the 13 competitive athletes with initial negative screening for SHD had VAs originating from the RV [20]. The innovative application of voltage-guided biopsy was an extremely sensitive approach to unmask concealed myocardial disease in athletes presenting with VAs: Indeed, all the patients with RV VAs and electrical scar displayed positive histological findings (5 ARVD, 4 myocarditis, 1 catecholamine excess cardiomyopathy), while all the three patients with the left-sided origin of the VAs showed myocarditis. Overall, the prevalence of genetic SHD, like ARVD, was present in 38% of the patients and acquired myocarditis in 54% of the study population.

A predominant RV involvement was prompted by Heidbuchel and colleagues, who recognized the presence of definite ARVD in 27 (59%) out of 46 high-endurance athletes (98% male, median age 31 years old, 80% cyclists), whilst signs of normal athletes’ heart and healthy coronary arteries were seen in 97% of the subjects studied (except for one case of bridging left anterior descending) [21]. 

Interestingly, Venlet et al. have recently analyzed fifty-seven competitive athletes (83% male, age 48 ± 16) with symptomatic VAs (ranging from non-sustained VT to VF/cardiac arrest) undergoing electrophysiological study and ablation [7]. Uni-bipolar endocardial and bipolar epicardial electroanatomical mapping (EAM) identified two patterns of distribution of RV scars: The first pattern is located at the level of the RV inflow (*n* = 46, 81%), the second instead at the level of the epicardial sub-pulmonic outflow tract (RVOT, *n* = 11, 19%). Notably, the first pattern was prevalently shown in non-endurance athletes with fewer hours of exercise per week. At the same time, the latter one was expressed exclusively in high-intensity endurance sports performers with more intense and more frequent sessions (30/46 (65%) vs. 11/11 (100%) *p* < 0.001). ECG featured LBBB morphology in all the VAs, which alongside the absence of LV involvement at the CMR, negative biopsy and genetic test, corroborated the conclusion that high-level endurance athletes exhibit a peculiar arrhythmogenic RV substrate. This pattern configured a unique structural adaptation named exercise-induced arrhythmogenic remodeling (EIAR), susceptible to ablation treatment.

Transient causes of myocardial changes associated with ventricular arrhythmias have been described anecdotally; for example, two high-intensity athletes with a history of ephedrine intake for doping purpose presented with LBBB inferior axis VAs (one case of sustained VT), corresponding to RVOT focal LGE, and biopsy findings were compatible with catecholamine excess cardiomyopathy [31].

## 5. Catheter Ablation of VA in Athletes: Indications, Outcomes, and Sport Eligibility

Unlike screening programs for SCD prevention and eligibility criteria, international guidelines lack specific indications about ablation of VAs in athletes, and the treatment essentially follows that recommended in non-athletes, described elsewhere [32,33,34,35,36,37,38]. Likewise, compared to the data regarding supraventricular tachycardias and atrial fibrillation, the experience of life-threatening VAs in athletes is by far less copious. Thus, some special considerations should be kept in mind and discussed with the patient and the sport cardiologists before pursuing any interventional treatment (Table 2). A recent document consensus stated that athletes with idiopathic monomorphic focal RVOT or interfascicular re-entrant VT should be treated early with the ablation instead of antiarrhythmics, and sports competition can be restarted after 3 months of negative follow-up [10]. On the contrary, patients with VT/VF and underlying genetic not reversible SHD should be restricted from intense physical activity, regardless of the response to ablation and should all receive an implantable cardiac defibrillator (ICD).

Venlet et al. dedicated the most insightful single-center prospective study to a cohort of athletes with a history of sustained VT or VF [7]. Thirty-one patients (54%) had a previously implanted ICD. Based on the EAM findings, the authors identified two distinct patterns of scar: One along the RV inflow (sub-tricuspid, group A)—variously ascribable to biopsy confirmed ARVD, myocarditis, or sarcoidosis—in 46 subjects, and one in the anterior sub-epicardial RVOT (group B). A re-entrant mechanism of the VT was noted in this latter group, and no complications were reported after the ablation. In spite of the absence of difference in VA burden before the ablation between groups, the post-ablation outcome was more favorable in group B, with a complete success of 91 vs. 57% (*p* = 0.034), and a recurrence at 27 months occurring in 0 vs. 50%.

Relevant information can be extrapolated by a single center registry of 47 patients with ARVD, 24 of which (51%) were former athletes (22/24 ex-endurance athletes) [39]. Half of the cohort underwent an endocardial ablation primarily, and a sequential epicardial or endo-epicardial ablation in case of lack of endocardial substrate or failure of the endocardial approach was endorsed in the remainder of patients. The ablation achieved complete acute success (non-inducibility) in 80% of the cases after the first ablation, and only one pericardial tamponade occurred. Freedom from the combined endpoint (sustained VT/VF, death, and heart transplant) was 47% at one year and 31% at five years after the index procedure, and the study endpoint improved after multiple procedures (63% and 45% respectively after 1 and 5 years). A statistically non-significant trend of arrhythmia-free survival was noted after multiple procedures in the combined endo-epicardial approach group after just one year compared to endocardial ablation only (75% vs. 53%, *p* = 0.058). Limited to the nature of the study, the authors did not find sport being a predictor of VT/VF recurrence, although all 24 competitive athletes discontinued physical activity at the time of the follow-up. 

A similar study led by Bai and coauthors, albeit on non-athletes with ARVD, carries informative conclusions regarding the overt benefit of the combined endo-epicardial approach in such patients. Indeed, VA recurrences and ICD shocks were drastically reduced in the group treated with endo-epicardial ablation compared to the control undergoing endocardial treatment alone (84.6% (22/26) vs. 52.2% (12/23), *p* < 0.029) for freedom from VAs. The necessity of continuous antiarrhythmic treatment was remarkably reduced as well in the group treated with the combined ablation (21.7% (5/23) vs. 69.2% (18/26), *p* < 0.001) [40]. 

Even though specific experiences are missing, VA ablation in patients with hypertrophic cardiomyopathy and monomorphic VT seems feasible, as proved by a multicenter case series of 10 non-athlete patients by Dukkipati and colleagues [41]. Epicardial substrate required additional epicardial ablation in 8 subjects; however, long-term results after a follow-up of 37.4 ± 16.9 months were promising, with a reduction of life-threatening VA-recurrence in 70% of the cohort. Likewise, Santangeli et al. showed that epicardial ablation accomplished an additional beneficial effect in 13 patients, six of which had failed the endocardial approach, with VT recurrence reported in only 3/10 of them (*p* = 0.6) [42]. 

Such reports, although not directly pertaining to VAs in athletes, can nonetheless be instrumental to guide the standard of care also for sport performers with the similar conditions.

Anecdotal, albeit significant cases, should also be considered. For instance, a young female athlete with ablation of RVOT-VT survived four years after that, before the exit for cardiac arrest due to post-mortem diagnosed ARVD [43]. Two cases of the successful Purkinje system VA have been separately described in athletes [16,44]. In one case where the follow-up was reported, the patient remained arrhythmia-free 15 months after the ablation. Table 3 reports a synopsis of the relevant studies described above. 

## 6. Conclusions and Knowledge Gaps

VAs are not always benign findings in athletes as they may indicate a cardiac substrate potentially impacting the patient’s life expectancy and professional career. Full non-invasive diagnosis is mandatory during the screening for eligibility, and both a 12-lead extended telemetry or exercise stress test with detailed imaging are critical to delineate the origin of the VAs, often featuring a non-ischemic source and arising from the RV rather than the LV. Moreover, EAM and biopsy have a significant diagnostic value in sensitively unmasking athlete’s heart’s specific arrhythmogenic conditions [7,20,31]. In contrast, catheter ablation of VAs in athletes is recommended in case of symptoms, focal monomorphic features—especially from the Purkinje system or from the RVOT—functional deterioration, or drug intolerance. Despite the modest and heterogeneous number of dedicated studies in this population, ablation seems to carry satisfactory results; nonetheless, given a greater prevalence of epicardial substrate, the procedure should be performed in tertiary centers where all the possible complications of the epicardial approach can be safely managed [25].

Further prospective studies with larger cohorts and extended follow-ups are needed to confirm the value of the ablation for the reduction of symptoms, the impact on the prevention of SCD, and superiority over medications or ICD.

## Figures and Tables

**Table 1:** Details of the research methods.

Primary Keyword	Logical Connectors	Secondary Keywords	Logical Connectors	Tertiary Keywords
- Sport	“AND”	- Ventricular arrhythmias - Ventricular tachycardia - Brugada - Hypertrophic cardiomyopathy - Dilated cardiomyopathy - Sudden cardiac death	“AND”	- Ablation - Catheter ablation - Electrophysiology - Heart
- Sport	“OR”	- Sport cardiology - Athlete - Exercise	“AND”	- Ventricular arrhythmias - Ventricular Tachycardia - Ablation - Catheter ablation - Electrophysiology
Brugada - Hypertrophic cardiomyopathy - Dilated cardiomyopathy - Arrhythmogenic right ventricular dysplasia	“AND”	- Ablation - Catheter ablation - Electrophysiology	“AND”	- Sport - Athlete - Exercise

**Table 2:** Considerations of catheter ablation of ventricular arrhythmias in athletes.

Pros	Cons
Reduction of symptoms	Short period of resting and detraining during the initial follow-up
Potential re-eligibility for competitive events after 3 months of negative follow-up	Temporary disqualification/non-eligibility for 3 months after the procedure
Avoidance of long-term treatment with antiarrhythmic drugs potentially acknowledged as doping	Higher risk of traumatic bleedings if started on anticoagulation/antiplatelets post-ablation
Benefit with a closer follow-up by both electrophysiologist and sport cardiologist	Avoidance with contact sports for 6 weeks in case of pericarditis post-ablation, especially after epicardial approach
Better understanding of the prevalence and features of arrhythmic substrate in this population	Acute procedural risks
Evidence of reduction of recurrence and ICD shocks	No evidence of reduction of SCD
	Costs

ICD: implantable cardiac defibrillator; SCD: sudden cardiac death.

**Table 3:** Principal studies of catheter ablation of ventricular arrhythmias in athletes.

Study	Type of Study	Year	Population, *n*	Type of VA	Critical Anatomical Substrate	Type of Ablation	Follow-Up Duration	Outcome
**Mathew** [39]	Retrospective non-randomized	2019	47 patients with ARVD (47% former endurance athletes) Age 44 ± 16 Male 38/47 (81%) Survived SCD 4 (9%)	VT/PVC (99%) LBBB superior axis (32%) LBBB inferior axis (23%) Polymorphic (44%) VF (1%)	RV inflow-subtricuspid (57% endocardial, 48% epicardial) RVOT (28% endocardial, 33% epicardial) RV apex (14% endocardial, 15% epicardial) LV (1% endocardial, 4% epicardial)	Endocardial+/− epicardial	50.8 months (18.6–99.2)	VT/VF recurrence from index ablation 37% after 1 year and 55% after 5 years
**Venlet** [7]	Prospective non-randomized	2017	57 athletes with VA history (47% active endurance athlete) Age 48 ± 16 Male 47/57 (83%) ARVD in 34/57 (60%) Survived SCD 6 (11%)	VT/PVC (92%) LBBB superior axis (21%) LBBB inferior axis (33%) LBBB both axis (57%) VF (8%)	RV inflow-subtricuspid (81%) Epicardial RVOT (19%)	Endocardial +/− epicardial	27 months (6–62)	VT/VF recurrence after index ablation in 40.3%
**Choung** [43]	Case report	2017	26 y/o African female athlete (runner) with tachycardia induced cardiomyopathy	VT, LBBB superior axis	RVOT	Endocardial	4 years	Recurrence of VA during her pregnancy Death for VF at the third trimester
**Reira** [44]	Case report	2009	32 y/o Caucasian cyclist	VT, RBBB left-superior axis (inter-fascicular VT)	Left anterior fascicle	Endocardial	N/A	Acute success
**Strohmer** [16]	Case report	2006	36 y/o male triathlete with a history of resuscitated cardiac arrest	VF/early coupled LBBB normal axis PVC	Mid-septal right ventricle	Endocardial	15 months	T wave over-sensing/inappropriate shock post-ablation requiring ICD-led revision

VA: ventricular arrhythmia; ARVD: Arrhythmogenic right ventricular dysplasia; LBBB: Left bundle branch block; LV: Left ventricle; PVC: Premature ventricular complex; RBBB: Right bundle branch block; RV: Right ventricle; RVOT: Right ventricular outflow tract; SCD: Sudden cardiac death; VF: Ventricular fibrillation; VT: Ventricular tachycardia.

## Data Availability

Not applicable.

## Abbreviations

CAD	coronary artery disease
CMR	cardiac magnetic resonance imaging
EAM	electroanatomical map
ICD	implantable cardiac defibrillator
LBBB	left bundle branch block
LGE	late gadolinium enhancement
PVCs	premature ventricular complexes
RBBB	right bundle branch block
SCA/SCD	sudden cardiac arrest/death
SHD	structural heart disease
VAs	ventricular arrhythmias
VF	ventricular fibrillation
VT	ventricular tachycardia
